# The influence of parental social support on academic burnout among vocational undergraduate students in Shandong Province, China

**DOI:** 10.3389/fpsyg.2026.1647498

**Published:** 2026-05-19

**Authors:** Haikuo Yu, Dong Yang

**Affiliations:** Department of Educational Administration, International College, Krirk University, Bangkok, Thailand

**Keywords:** academic burnout, academic self-efficacy, parental social support, Shandong Province, vocational undergraduate students

## Abstract

Academic burnout is common among Chinese vocational undergraduate students, where high tuition fees, low motivation, and emotional exhaustion pose frequent challenges. Parental social support and academic self-efficacy have been shown to alleviate these challenges, and gender differences may affect their impact. This study explores the mediating role of academic self-efficacy in the association between parental social support and academic burnout. It also analyzes the moderating role of gender in the association between parental social support and academic self-efficacy among vocational undergraduate students in Shandong Province, China. In total, 563 vocational undergraduate students from three vocational undergraduate colleges in Shandong Province were recruited for this study. The gender distribution of the sample was relatively balanced (50.1% male and 49.9% female). Participants were predominantly aged between 18 and 21 years, and the largest proportion were first-year students, followed by fourth-year, second-year, and third-year students. Structural equation modeling was employed to establish both measurement and structural models. Our findings show that parental social support was negatively associated with students’ academic burnout. In addition, academic self-efficacy partially mediated the association between parental social support and academic burnout. The association between parental social support and academic self-efficacy was significantly stronger among male than female students. These findings suggest that vocational undergraduate colleges should provide targeted academic guidance, especially for female students, to strengthen academic confidence and help reduce academic burnout.

## 1 Introduction

Vocational education is a distinctive educational model within international higher education, designed specifically to equip students with practical skills and knowledge tailored to specific industries. In the context of Chinese higher education, the National Vocational Education Reform Implementation Program was enacted in February 2019, calling for the establishment of private undergraduate vocational education in China based on private Chinese colleges (Wang, 2023). A significant characteristic of this type of education model is higher tuition fees, which lead to higher education expenses. In China, parents typically provide educational support as a form of investment, with the aim of ensuring their children’s success and preparing them to become the primary providers for the family (Louie, 2001). Due to the unique characteristics of this educational model, the financial pressure on the parents of vocational undergraduate students is among the highest across all forms of higher education (Wang, 2023; Xiong, 2011). Therefore, parental social support plays a crucial role in students’ academic careers, encompassing both verbal and nonverbal information or advice delivered in the form of emotional, appreciative, decision-making, and material assistance (Procidano and Heller, 1983). High levels of parental social support have been shown to mitigate students’ academic pressure, psychological distress, and symptoms of burnout in learning contexts (Ye et al., 2021).

Academic burnout refers to the burnout that students experience, characterized by emotional exhaustion and apprehension regarding their studies (Schaufeli et al., 2002). In vocational undergraduate colleges, students’ academic burnout can result in high absenteeism, elevated dropout rates, and a decline in academic performance (Jacobs and Dodd, 2003). Studies have revealed that vocational undergraduate students experience academic burnout characterized by emotional exhaustion, cynicism, and feelings of inefficacy (Schaufeli et al., 2002). This is primarily due to the inability of students to focus on their studies because of the financial burden of part-time work and the inability to study adequately owing to a lack of confidence in their study content (Joo et al., 2008; Ma, 2024). Such situations leading to academic burnout are encountered not only by international students but also by Chinese vocational undergraduate students (Tang and Osman, 2023; Xia et al., 2025). However, these situations can be mitigated if students receive psychological and financial support from their parents. Moreover, previous studies have noted that parental social support can help students reduce psychological distress (e.g., academic burnout) (Chyu and Chen, 2024). Existing studies have predominantly centered on the direct association between parental social support and academic burnout (Liu et al., 2023; Wang et al., 2021), while the psychological mechanisms underlying this association—particularly the specific pathway through which this association operates in the vocational undergraduate education setting—have yet to be comprehensively explored.

Academic self-efficacy refers to the belief or judgment an individual holds regarding their ability to successfully complete specific tasks and achieve learning goals in academic activities (Artino, 2012; Zimmerman, 2000). Prior research has suggested a broader “social support–academic self-efficacy–outcome” pathway (Green et al., 2024; Zhang and Jin, 2016). Building on this line of research, academic self-efficacy may serve as a potential mechanism linking parental social support to academic burnout. Academic self-efficacy theory suggests that encouragement and support from others can serve as important sources of students’ efficacy beliefs in academic contexts. Such encouragement may strengthen students’ confidence in dealing with academic demands and help them cope with difficult situations (Artino, 2012). However, although existing theory and prior research suggest that self-efficacy may play an important mediating role, this mechanism has not been sufficiently examined in the specific association between parental social support and academic burnout, particularly in the context of Chinese vocational undergraduate education. Accordingly, the current study applies the “social support–academic self-efficacy–outcome” pathway to this specific educational setting and examines whether academic self-efficacy helps explain the association between parental social support and academic burnout.

Gender typically refers to the socially constructed roles, behaviors, and identities associated with women, men, and gender-diverse individuals within specific cultural contexts (Mazzuca et al., 2020). From the perspective of gender socialization, family processes, as reflected in the Gendered Family Process Model, may shape how parental social support is received, interpreted, and internalized across gender groups (Endendijk et al., 2018; Zosuls et al., 2011). This may in turn help explain why parental social support may not be translated into academic self-efficacy to the same degree across gender groups. This possibility may be particularly relevant in the context of Chinese vocational undergraduate education with high tuition burdens, especially in Shandong, where Confucianism has long shaped gendered educational expectations within families (Qing, 2020; Xiong, 2011). Accordingly, within the broader “social support–academic self-efficacy–outcome” pathway (Green et al., 2024; Zhang and Jin, 2016), the present study extends this line of inquiry to gender by examining whether the association between parental social support and academic self-efficacy differs across gender groups.

Building on the preceding analysis, this study focuses on the associations among parental social support, academic self-efficacy, and academic burnout, while further exploring the moderating role of gender in the parental social support–academic self-efficacy link. By examining these associations, the present study may help educational administrators better understand the role of parental social support in academic burnout among Chinese vocational undergraduate students and support the development of more targeted interventions for students who may be at risk of academic burnout, thereby helping to prevent its negative consequences.

## 2 Research hypothesis and hypothetical model

External factors contributing to academic burnout include demanding and stressful academic tasks as well as insufficient parental support (Liu et al., 2023). Prior studies have shown that parental social support is associated with lower levels of students’ psychological distress and academic burnout (Kim et al., 2018; Wang et al., 2021). As an important family resource, parental social support may help students maintain emotional stability, cope more effectively with academic challenges, and reduce negative experiences such as anxiety, exhaustion, and burnout. From the perspective of social cognitive theory (Bandura, 2001), supportive environmental resources can facilitate students’ positive adaptation by strengthening their confidence in dealing with academic demands and buffering the effects of stress. Accordingly, parental social support may be negatively associated with academic burnout among vocational undergraduate students in Shandong Province, China. On this basis, this study first puts forward a direct association between parental social support and academic burnout:

*H1*: Parental social support is negatively associated with academic burnout among vocational undergraduate students in Shandong Province, China.

Existing studies have suggested that academic self-efficacy may serve as a potential mediating mechanism between social support and outcomes (Green et al., 2024; Zhang and Jin, 2016), implying that the association between parental social support and academic burnout in this study may not be exclusively direct; an indirect association mediated by academic self-efficacy may exist. According to academic self-efficacy theory, parental encouragement and emotional reassurance can reinforce students’ confidence in their ability to complete academic tasks and deal with academic difficulties and challenges within the higher education context (Artino, 2012). Accordingly, parental social support may shape academic outcomes not only through the provision of external assistance, but also by cultivating students’ beliefs regarding their own academic capabilities.

First, previous research has found that parental social support is positively associated with academic self-efficacy. For instance, Karademas (2006) examined the association between social support, perceived stress, self-efficacy, and life satisfaction among 94 freshmen and found that parental social support had a positive effect on self-efficacy. Narayanan and Onn (2016) surveyed 377 freshmen at a public university in Malaysia and found that both social support and self-efficacy were significant predictors of resilience, with one study demonstrating that social support positively influenced self-efficacy. Ren et al. (2020) measured social support and self-efficacy using the Social Support Scale and Self-Efficacy Scale in a sample of 2,341 Chinese adolescents. The findings indicated that self-efficacy acted as an important mediator in the association between social support and physical activity. They also found that social support had a positive effect on self-efficacy. These associations can be interpreted through social cognitive theory (Bandura, 2001), which posited that support from social relationships (e.g., family and friends) could help individuals increase their self-efficacy and cope with stress when facing challenges.

Second, academic self-efficacy has been linked to academic burnout. For example, Charkhabi et al. (2013) explored the Self-Efficacy and Academic Burnout Scales among 233 undergraduate students and found that students with high self-efficacy exhibited a greater sense of competence and generated more positive emotions, avoiding academic burnout resulting from a lack of competence. Rahmati (2015) examined 120 students at the Allameh Tabataba’i University, who completed questionnaires on both academic burnout and self-efficacy. The results revealed that students with high self-efficacy were more motivated to complete tasks and developed more positive emotions, avoiding academic burnout due to lack of competence. Ma (2024) investigated 580 Chinese learners of English as a Foreign Language (EFL) recruited from different universities in Henan Province. The study assessed academic self-efficacy and motivation, both of which were found to be strong negative predictors of academic burnout among Chinese EFL learners. These associations can also be interpreted through the self-determination theory (Ryan and Deci, 2000), whereby an individual’s autonomy, sense of relatedness, and sense of competence were key factors in maintaining positive motivation. Students with high self-efficacy have a stronger sense of competence that sustains intrinsic motivation and reduces academic burnout, which could be due to feeling out of control over academic tasks (Singh et al., 2021).

In addition to the partial associations between parental social support, academic self-efficacy, and academic burnout, existing studies have proposed a model of “social support–academic self-efficacy–outcome”. For example, social support has been shown to influence learning outcomes through self-efficacy (Labrague, 2024). Furthermore, social support enhanced confidence in completing academic requirements, thereby increasing academic self-efficacy (Saefudin et al., 2021). A cross-sectional quantitative study was conducted with a sample of 80 final-year undergraduate students using a questionnaire comprising three scales: the Generalized Anxiety Disorder-7, Academic Self-Efficacy Scale, and Multidimensional Scale of Perceived Social Support. The results showed that academic self-efficacy fully mediated the association between perceived social support and student anxiety (Laksmiwati and Tondok, 2023). Zhang et al. (2024) explored the mediating role of academic self-efficacy in the association between social support and procrastination behaviors among college students in a Chinese higher education institution. A survey of 1,379 university students revealed a significant negative correlation between social support and procrastination, mediated by academic self-efficacy. Based on the above analyses, the association between parental social support and academic burnout may not be exclusively direct, but may also function through academic self-efficacy as a key cognitive mechanism. Therefore, the following research hypothesis is proposed:

*H2*: Academic self-efficacy of vocational undergraduate students in Shandong Province mediates the association between parental social support and academic burnout.

Existing research with university students also suggests that parental inputs may not be associated with self-efficacy in entirely gender-neutral ways. For example, parent-related inputs have been found to shape self-efficacy-relevant career processes in undergraduate samples, and parent-related influences have also been shown to relate differently to career decision-making self-efficacy for male and female college students (Garcia et al., 2012; Lease and Dahlbeck, 2009). Although these findings concern career-related rather than academic self-efficacy, they still suggest that the parental input–self-efficacy link may vary across gender groups. From the perspective of gender socialization, family processes, as reflected in the Gendered Family Process Model, may influence the meaning students attach to parental support across gender groups (Endendijk et al., 2018; Zosuls et al., 2011). Within families, parental support is often accompanied by expectations about children’s future roles and responsibilities (Endendijk et al., 2018; Halpern and Perry-Jenkins, 2016). When those expectations differ for sons and daughters, the same support may be understood differently and may therefore be less readily translated into academic self-efficacy for some students than for others (Endendijk et al., 2018; Halpern and Perry-Jenkins, 2016).

In the context of Chinese vocational undergraduate education, which is characterized by high tuition burdens (Xiong, 2011), gendered family expectations may further shape how parental support is translated into academic self-efficacy. This possibility may be especially pronounced in Shandong Province, China, where Confucian traditions continue to influence family role expectations and sons may be more likely than daughters to be associated with future financial responsibility (Qing, 2020). In such a context, parental social support directed toward male students may be more readily interpreted as recognition of competence and future potential (Murphy et al., 2011; Wang et al., 2020). Consequently, when male and female students receive comparable parental social support, male students may be more likely to internalize that support as confidence in their academic abilities, whereas the same support may be translated into academic self-efficacy to a comparatively lesser degree among female students (Huang, 2013; Wang et al., 2020). Accordingly, the positive association between parental social support and academic self-efficacy may be stronger for male students than for female students. Therefore, the following hypothesis is proposed:

*H3*: Gender moderates the association between parental social support and academic self-efficacy among vocational undergraduate students in Shandong Province, China, such that the positive association between parental social support and academic self-efficacy is stronger for male students than for female students.

**Figure 1:**
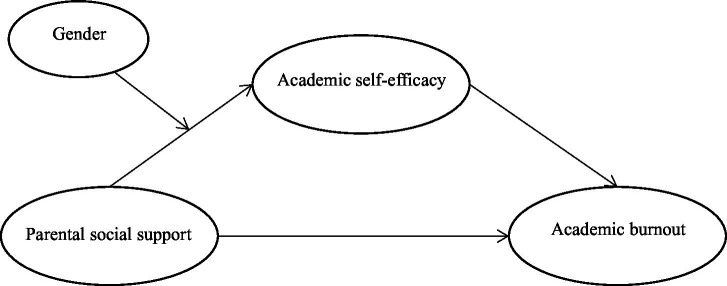
Research framework diagram.

The hypothesized model used in this study (Figure 1) was constructed based on previous research and relevant theories. Parental social support was expected to be negatively associated with academic burnout and positively associated with academic self-efficacy. Academic self-efficacy was expected to be negatively associated with academic burnout and to mediate the association between parental social support and academic burnout. Gender was included as a moderator of the association between parental social support and academic self-efficacy.

## 3 Materials and methods

### 3.1 Participants

Shandong Province, a major educational province in China, has a large population and a relatively developed economy and has taken the lead in piloting reforms to vocational undergraduate education. Therefore, vocational undergraduate students from Shandong Province were selected for this study. To ensure gender balance, a purposive sampling method was employed to select full-time students from vocational undergraduate colleges in Shandong Province who needed to rely on parental financial support. Students who were chronically absent from class, received psychological support, or engaged in long-term and intensive part-time work were excluded to ensure data validity. From June 1 to June 30, 2024, 800 online questionnaires were evenly distributed to students at three vocational undergraduate colleges by vocational undergraduate counselors in Shandong Province. The researchers explained the purpose of the study to the participants and asked them to provide written informed consent. Participation was voluntary, and all data were kept confidential. Notebooks were gifted to each participant to encourage participation. A final valid sample of 563 participants was obtained, and all data were provided by vocational undergraduate students in the Shandong Province. The demographic characteristics of the participants are shown in Table 1.

**Table 1:** Sample demographics.

Type	Group	*N*	%
Gender	Male	282	50.1
	Female	281	49.9
Age	Under 18	58	10.3
	18–19	287	51.0
	20–21	130	23.1
	22 and above	88	15.6
Grade	Freshman	268	47.6
	Sophomore	102	18.1
	Junior	67	11.9
	Senior	126	22.4
Availability of part-time jobs	Yes	99	17.6
	No	464	82.4

### 3.2 Materials

This study measured three interrelated concepts, which were developed from theory and supported by research, rated on a Likert 5-point scale ranging from 1 (strongly disagree) to 5 (strongly agree), defined as follows:

1. Parental social support. Parental social support refers to the support provided by parents encompassing verbal and non-verbal information or advice delivered in the form of emotional, appreciative, decision-making, and material assistance (Procidano and Heller, 1983). The level of parental social support was measured using eight parent-related support items from Yeh et al. (2008), as cited in Yang et al. (2023).
2. Academic self-efficacy. Academic self-efficacy refers to the beliefs or judgements an individual holds about their ability to successfully complete specific tasks and achieve learning goals in academic activities (Zimmerman, 2000; Artino, 2012). Academic self-efficacy was measured using a 5-item questionnaire (Nielsen et al., 2018).
3. Academic burnout. Academic burnout refers to the burnout that students experience in their studies, where they feel emotionally exhausted and lack confidence (Schaufeli et al., 2002). Academic burnout was measured using a 15-item questionnaire (Schaufeli et al., 2002).

### 3.3 Analytical method

This study employed AMOS software (version 21.0) to conduct a confirmatory factor analysis (CFA) to assess the reliability and validity of the instrument. Several evaluation metrics were used, including item model fit, item factor loadings, error variances, inter-factor correlations (Byrne, 2010), sample normality (Hair et al., 2010), scale internal consistency (Streiner, 2003), convergent validity (Hair et al., 2010), and discriminant validity (Torkzadeh et al., 2003). The items retained through the CFA were used to construct mediation models in AMOS, and bootstrap estimation was performed to assess the mediation effects (Hashmi et al., 2021). Based on the mediating effect, the potential moderating effects in the model were further tested using a multi-group analysis (Rahman et al., 2018).

## 4 Results

### 4.1 Reliability and validity

First, we constructed a multifactor oblique measurement model based on three variables—parental social support, academic self-efficacy, and academic burnout—consisting of 28 questionnaire items (Table 2). An examination of the reliability and validity indicators and their criteria in the measurement model (summarized in Table 3) revealed that the model fit for the 28 questionnaire items was good, demonstrating reasonable internal consistency and construct validity. Therefore, all 28 items were used to construct the structural model.

**Table 2:** Question items and related parameters.

Variable	Questionnaire items	Factor loading
Parental social support	1. When I need help, my parents help me analyze the ins and outs and possible reasons.	0.705
	2. My parents make me feel safe and happy.	0.711
	3. My parents make me feel that no matter what decision I make, they will support me.	0.710
	4. My parents accompany me at dinner, talk to me, or perform other leisure activities with me so as to make me forget my troubles.	0.714
	5. My parents listen to me intently when I am describing a situation or specifying my complaints; this makes me feel like they understand me.	0.696
	6. My parents encourage me and make me feel that I have to work harder to solve my problems.	0.715
	7. My parents give me money or other material assistance when I need help.	0.716
	8. My parents need me, and this makes me feel I am important to them.	0.710
Academic self-efficacy	9. I generally manage to solve difficult academic problems if I try hard enough.	0.726
	10. I know I can stick to my aims and accomplish my goals in my field of study.	0.723
	11. I will remain calm in my exam because I know I will have the knowledge to solve the problems.	0.687
	12. I know I can pass the exam if I put in enough work during the semester	0.685
	13. The motto ‘If other people can, I can too’ applies to me when it comes to my field of study.	0.774
Academic burnout	14. I feel emotionally drained by my studies.	0.716
	15. I feel used up at the end of a day at university.	0.707
	16. I feel tired when I get up in the morning and I have to face another day at the university.	0.753
	17. Studying or attending a class is really a strain for me.	0.725
	18. I feel burned out from my studies.	0.737
	19. I have become less interested in my studies since my enrollment at the university.	0.709
	20. I have become less enthusiastic about my studies.	0.725
	21. I have become more cynical about the potential usefulness of my studies.	0.728
	22. I doubt the significance of my studies.	0.753
	23. I can effectively solve the problems that arise in my studies.	0.740
	24. I believe that I make an effective contribution to the classes that I attend.	0.757
	25. In my opinion, I am a good student.	0.752
	26. I feel stimulated when I achieve my study goals.	0.717
	27. I have learned many interesting things during the course of my studies.	0.710
	28. During class I feel confident that I am effective in getting things done.	0.762

Parental social support (questionnaire items 1–8), academic self-efficacy (questionnaire items 9–13), and academic burnout (questionnaire items 14–28).

**Table 3:** Reliability and validity indicators.

Indicators	Reliability and validity indicators
Model fit	*χ*^2^ = 383.919, *p* < 0.001, *χ*^2^/df = 1.106, GFI = 0.955, CFI = 0.995, NFI = 0.954, IFI = 0.995, PNFI = 0.876, RMSEA = 0.014
Factor loading	0.696–0.716, 0.685–0.774, 0.707–0.762 (all > 0.50)
Error variance	19.098, 21.711, 31.055% (all were positive and significant)
Correlations	−0.48 ≤ values ≤ 0.44
Univariate normality	skewness (−0.911, −0.541, 1.272) (all absolute value) < 3, kurtosis (1.318, 0.285, 1.183) (all absolute value) < 10
Multivariate normality	Mardia’ coefficients 30.987 < 840 [calculated as p(p + 2), where p is the number of measurement items]
Item consistency	Cronbach’s *α*–0.890, 0.842, 0.946 (all > 0.8)
Convergent validity	CR–0.890, 0.843, 0.946 (all > 0.7)
Discriminant validity	Confidence interval [0.335, 0.529], [−0.572, −0.379], [−0.515, −0.310] (all were excluding 0 and significant)

Variables from left to right are parental social support, academic self-efficacy, academic burnout. *** *p* < 0.001, ** *p* < 0.01, * *p* < 0.05. CR, composite reliability; CFI, comparative fit index; GFI, goodness-of-fit index; NFI, Normed Fit Index; IFI, Incremental Fit Index; PNFI, Parsimony-Adjusted Normed Fit Index; RMSEA, root mean square error of approximation; *χ*^2^, chi-square; *χ*^2^/df, normed chi-square.

In addition, common method bias was examined using Harman’s (1976) single-factor test. The first unrotated principal component accounted for 37.715% of the total variance, which was below the 40% threshold, suggesting that common method bias was not a serious concern in this study.

### 4.2 The mediating effects of academic self-efficacy

**Figure 2:**
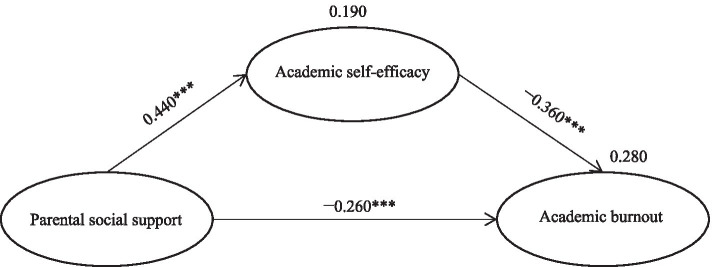
Total mediating model ***p < 0.001.

To test H2, we constructed a mediation model involving parental social support, academic self-efficacy, and academic burnout using the 28 questionnaire items retained from the measurement model. The mediation model demonstrated good model fit (*χ*^2^ = 383.919, *p* < 0.001, *χ*^2^/df = 1.106, GFI = 0.955, CFI = 0.995, RMSEA = 0.014) and reasonable factor loadings (ranging from 0.690 to 0.760). As shown in the mediation effect plot in Figure 2, we first modeled the direct effect of parental social support on academic burnout (parental social support → academic burnout, *γ* = −0.260, *p* < 0.001). Therefore, H1 was supported. Due to the addition of the mediating variable, parental social support (*γ* = −0.260, *p* < 0.001) and academic self-efficacy (*γ* = −0.360, *p* < 0.001) jointly explained academic burnout (*R*^2^ = 28%). Parental social support independently explained academic self-efficacy (*γ* = 0.440, *p* < 0.001, R^2^ = 19%). As the constructed model presented a potential mediating structure, we assessed the direct, indirect, and overall effects of parental social support on academic burnout using the bootstrap method. As shown in Table 4, there was an indirect effect between parental social support and academic burnout caused by academic self-efficacy (*θ* = −0.160, *p* < 0.001). This indirect effect accounted for part of the total effect between parental social support and academic burnout (*θ* = −0.420, *p* < 0.001); thus, the direct effect between parental social support and academic burnout was only partially retained (*θ* = −0.260, *p* < 0.001). These results confirmed H2 and indicated that the constructed mediation model was partial mediation.

**Table 4:** The mediating effect between parental social support and academic burnout.

Mediating effect	Value	Bias-corrected percentile method 95% confidence interval	
		Lower bound	Upper bound
Total effect	−0.42***	−0.515	−0.310
Direct effect	−0.26***	−0.361	−0.155
Indirect effect	−0.16***	−0.227	−0.107

*** p < 0.001.

### 4.3 The moderating effect of gender

To test H3, we divided the sample into male (*n* = 282) and female (*n* = 281) groups and conducted a multigroup analysis based on the path of the mediation model. First, the free multigroup model demonstrated acceptable model fit (*χ*^2^ = 1143.972, *p* < 0.05, *χ*^2^/df = 1.099, GFI = 0.936, CFI = 0.994, RMSEA = 0.009) (Byrne, 2010). Moreover, the factor loadings in the model estimates (male group: 0.656–0.799; female group: 0.600–0.779) were reasonable. In the free model estimation (i.e., the models for the male and female groups were estimated independently without any constraints), the path “parental social support → academic self-efficacy” was positively predicted for both males (*γ* = 0.610, *p* < 0.001) and females (*γ* = 0.300, *p* < 0.001).

**Figure 3:**
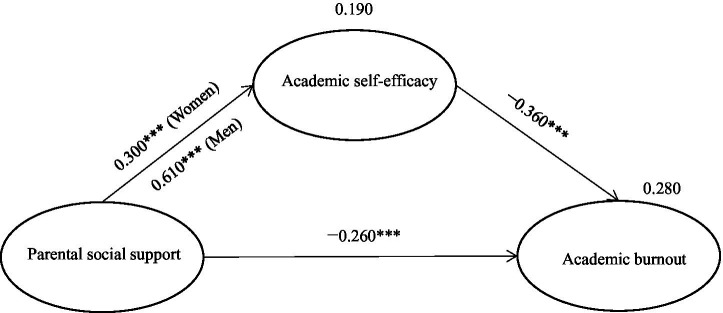
Mediating model for the male and female groups ***p<0.001.

We hypothesized that the paths of “parental social support → academic self-efficacy” in the mediation model were equal for men and women, that was, “parental social support → academic self-efficacy (men) = parental social support → academic self-efficacy (women)” (Byrne, 2010; Paulssen et al., 2014). As shown in Table 5, a comparison of the two model estimates revealed a difference in chi-square values between the free and restrictive model estimations (△*χ*^2^ = 8.936, *p* = 0.003 < 0.01; free model estimation: *χ*^2^ = 1143.972, *p* < 0.05, *χ*^2^/df = 1.099, GFI = 0.936, CFI = 0.994, RMSEA = 0.009; restrictive model estimation: *χ*^2^ = 1152.908, *p* < 0.05, *χ*^2^/df = 1.106, GFI = 0.935, CFI = 0.993, RMSEA = 0.010). Thus, the path equality constraint assumed in the restrictive model estimation was not supported. We further examined pairwise parameter comparisons in the free model estimation and found that there was a statistically significant difference in the “parental social support → academic self-efficacy” (*Z* = 2.958, *p* < 0.01) pathway between the male and female groups (greater for males than for females). This difference is reflected in the fact that the “parental social support → academic self-efficacy” association was much stronger in the male group (*γ* = 0.610, *p* < 0.001) than in the female group (*γ* = 0.300, *p* < 0.001) (as shown in Figure 3; Table 5). Therefore, H3 was supported, indicating that gender moderated the association between parental social support and academic self-efficacy in the mediation effect of parental social support, academic self-efficacy, and academic burnout.

**Table 5:** The moderating effect between parental social support and academic self-efficacy.

Parametric			Difference
*χ*^2^ value	Free model estimation	1143.972***	8.936**
	Restrictive model estimation	1152.908***	
Default model path value	Men	0.610***	2.958**
	Women	0.300***	

*** p < 0.001, **p < 0.01.

**Figure 4:**
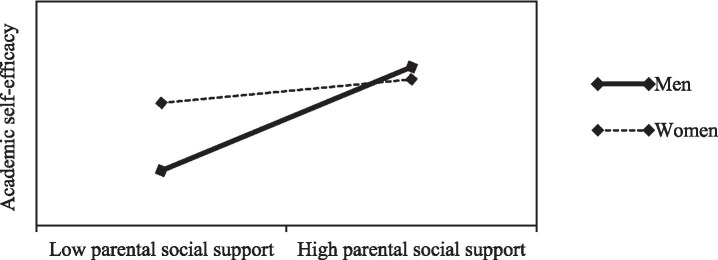
Moderating effect of gender on the association between parental social support and academic self-efficacy.

The simple slope analysis further revealed (as shown in Figure 4) that parental social support positively predicted academic self-efficacy for both male and female vocational undergraduate students, with a steeper regression slope observed in the male group, indicating that gender moderated the strength of the effect within this pathway. More precisely, as parental social support rose from low to high levels, the magnitude of increase in academic self-efficacy was significantly more pronounced among male students than among their female counterparts. This finding confirms that parental social support exerts a more facilitative effect on academic self-efficacy for male students.

## 5 Discussion

In this study, we investigated the direct association between parental social support and academic burnout, and examined whether this association aligned with the proposed H1. The results supported H1, showing that parental social support was significantly and negatively associated with academic burnout. This study explored the mediating role of academic self-efficacy in the association between parental social support and academic burnout. Furthermore, we tested whether this mediated association differed by gender among vocational undergraduate students in China, as hypothesized in H2 and H3. The results for H2 suggested that academic self-efficacy partially mediated the association between parental social support and academic burnout. Meanwhile, the results for H3 indicated that the association between parental social support and academic self-efficacy differed between male and female vocational undergraduate students in China. In this context, parental social support was more strongly associated with academic self-efficacy among male students.

First, the findings for H1 suggested that parental social support was negatively associated with academic burnout among vocational undergraduate students. This finding aligns with recent studies demonstrating that parental social support generally acts as a critical protective factor against academic burnout (Ye et al., 2021; Zhang et al., 2025). Relative to their peers in general undergraduate education, vocational undergraduate students frequently encounter more intensive practice-oriented training and heavier employment-related pressure. Accordingly, parental social support in this group, including encouragement and advice, may help reduce students’ academic burden, foster a more optimistic attitude, mitigate negative emotions such as anxiety, and improve their self-confidence, which in turn may reduce their academic burnout (Ye et al., 2022).

Second, the results for H2 suggested that academic self-efficacy partially mediated the association between parental social support and academic burnout among Chinese vocational undergraduate students. This result is also consistent with the broader “social support–academic self-efficacy–outcome” pathway (Green et al., 2024; Zhang and Jin, 2016). Building on the direct association identified in H1, this finding can be understood in light of academic self-efficacy theory, which suggests that encouragement and emotional reassurance from significant others can strengthen students’ confidence in dealing with academic tasks and difficulties (Artino, 2012). This interpretation is particularly relevant in the context of Chinese vocational undergraduate education, where practice-oriented training, career preparation, and relatively high educational costs may increase students’ dependence on family support (Li et al., 2023; Wang, 2023; Xiong, 2011). In this context, parental social support may be associated with lower academic burnout not only directly, as indicated by H1, but also indirectly by strengthening students’ confidence in managing academic demands through academic self-efficacy.

Third, the results for H3 showed that gender moderated the association between parental social support and academic self-efficacy. Specifically, the positive association between parental social support and academic self-efficacy was stronger among male students than among female students. This finding can be interpreted from the perspective of gender socialization. As suggested by the Gendered Family Process Model, family processes may shape the meanings that students attach to parental support across gender groups (Endendijk et al., 2018; Zosuls et al., 2011). In the context of Chinese vocational undergraduate education in Shandong Province, parental support may not be interpreted simply as a general supportive resource, but may also carry gendered expectations regarding students’ future roles, responsibilities, and academic development. Given the continuing influence of Confucian family role expectations in this context (Qing, 2020; Xiong, 2011), support directed toward male students may be more readily interpreted as recognition of competence and future potential, which may help explain why parental social support was more strongly associated with academic self-efficacy among male students than among female students (Murphy et al., 2011; Wang et al., 2020).

More specifically, within this framework, parental support becomes educationally meaningful through the family expectations attached to it. When parents provide different forms of educational support, students may interpret such support not only as care, but also as an implicit message about what the family expects them to become. In a context where sons may be more closely associated with future economic responsibility within the family, parental support for male students may be more likely to carry a competence-affirming meaning (Murphy et al., 2011; Wang et al., 2020). In other words, male students may interpret parental support as a signal that their families believe in their academic capability and expect them to assume future responsibilities. Such a meaning may make parental support more readily internalized as confidence in academic ability, thereby strengthening its association with academic self-efficacy.

By contrast, parental support for female students may also be beneficial, but its meaning may be less directly tied to competence affirmation. Within the same gender socialization framework, daughters may be more likely to receive support within a family script that emphasizes care, protection, relational responsibility, and future family roles (Endendijk et al., 2018; Halpern and Perry-Jenkins, 2016). In this case, parental support may be understood as emotional concern rather than as a clear message that the family expects them to develop academic competence and assume future achievement-related responsibility. As a result, even when female students receive comparable parental support, the support may be less likely to function as a direct signal of academic capability and may therefore be less strongly translated into academic self-efficacy. This interpretation does not imply that female students benefit less from parental support in general, nor does it suggest any innate difference in academic ability. Rather, the stronger association observed among male students may reflect gendered differences in the meanings attached to parental support and in the process through which such support is internalized as academic self-efficacy.

Overall, the findings of this study should be understood in relation to the distinctive psychological characteristics and academic adaptation mechanisms of vocational undergraduate students compared with students in general academic undergraduate education. Consistent with existing scholarly literature, vocational undergraduate students, when compared to their peers in general academic undergraduate education, tend to confront more prominent career and employment orientations, greater practical and employment-related pressures, as well as lower perceived social prestige and social status (Liu et al., 2022; Lu and Wang, 2023). Prior research on higher vocational education students has further demonstrated that factors including social support, academic hope, professional identity, and employment expectations are strongly correlated with their academic adaptation and developmental outcomes (Huang et al., 2025; Zhu et al., 2025). On this basis, among this student population, the association between parental social support and academic burnout may be more likely to operate through academic self-efficacy. This suggests that the same underlying psychological mechanism may differ in both its manifestation and explanatory power across different types of higher education, which represents a contextualized contribution of this study.

## 6 Theoretical implications

The theoretical contribution of this study lies in refining the broader “social support–academic self-efficacy–outcome” pathway by specifying social support as parental social support and situating the pathway in the context of Chinese vocational undergraduate education. In doing so, the present study not only provides further support for the direct association between parental social support and academic burnout, but also draws on academic self-efficacy theory to explain the indirect association between them. Specifically, parental encouragement may strengthen students’ confidence in dealing with difficulties, which in turn may be associated with lower academic burnout (Artino, 2012). For Chinese vocational undergraduate students, academic burnout remains a salient academic challenge (Tang and Osman, 2023; Xia et al., 2025). In this context, this indirect effect helps clarify how parental social support may be linked to academic burnout through academic self-efficacy.

The gender-related findings further refine the broader “social support–academic self-efficacy–outcome” pathway by showing that the association between parental social support and academic self-efficacy may differ across gender groups. Specifically, the positive association between parental social support and academic self-efficacy was stronger among male students than among female students. This suggests that parental social support may not be translated into academic self-efficacy to the same degree across gender groups. From the perspective of gender socialization and the Gendered Family Process Model, this difference may reflect the gendered meanings attached to parental support within family processes, particularly in relation to students’ future roles, responsibilities, and academic development (Endendijk et al., 2018; Zosuls et al., 2011). In the present study, because the association between parental social support and academic self-efficacy was stronger among male students, the indirect pathway from parental social support to academic burnout through academic self-efficacy may also be stronger for male students than for female students. Therefore, the mediating effect identified in this study and its possible gender-related differences may provide a useful reference for future scholars seeking to develop a more differentiated understanding of the broader “social support–academic self-efficacy–outcome” pathway.

## 7 Practical implications

Vocational education specializes in preparing students for practical skills and industry-specific knowledge. A significant characteristic of this educational model is high tuition fees; thus, students face higher educational expenses. Academic burnout can lead to high absenteeism, high dropout rates, and declining academic performance among vocational undergraduate students (Jacobs and Dodd, 2003). First, vocational undergraduate colleges, as a crucial part of the Chinese higher education system, are dedicated not only to providing vocational skills education but also to enhancing students’ comprehensive quality and employment competitiveness (Wang, 2023). Many vocational undergraduate colleges alleviate financial pressure by partnering with businesses that offer paid internships and part-time opportunities for students to gain practical experience in school (Wang, 2023; Xiong, 2011). Based on the results of this study, the association between parental social support and academic self-efficacy was weaker among female students than among male students. This finding should not be interpreted as lower academic ability among female students, but may suggest that parental support is less directly internalized as academic confidence in this group. Therefore, it may be more challenging for female students to meet the demands of their academic studies. To address this issue, schools should provide targeted academic guidance for female students. This could include establishing dedicated counseling programs for female students and conducting lectures to build academic confidence. These initiatives should focus on helping female students strengthen their academic confidence and cope with academic challenges more effectively. In addition, it is crucial that schools adopt gender-sensitive strategies when offering opportunities for practical experience such as internships and part-time employment. Female students could specifically benefit from more tailored opportunities in these areas, which would allow them to gain valuable work experience and develop professional skills while simultaneously enhancing their academic confidence. Providing internships or part-time positions with flexible working hours and focusing on sectors that actively promote female participation can further empower female students, strengthening their confidence and better preparing them for their future careers.

## 8 Limitations and future directions

First, this study focused on vocational undergraduate students in Shandong Province, which may have resulted in regional and cultural contextual limitations. Given that Shandong is a region with unique educational traditions and distinct cultural features, the generalizability of the current findings to other regions, cultural contexts, or other types of higher education institutions requires further empirical validation. In addition, this study was based on parental social support and did not distinguish between paternal and maternal support or account for the absence of either source of support due to factors such as parental divorce or bereavement. As such, this study has inherent limitations in capturing the complexity of family structures, and was unable to fully delineate the differential impacts of parental social support on academic burnout across varied family contexts. Therefore, future research should be expanded to other regions and types of universities to assess the generalizability of the model. Meanwhile, subsequent research may further disentangle the specific roles of paternal and maternal support, and in combination with diverse family structures such as single-parent families, reconstituted families, and grandparent-led caregiving arrangements, conduct an in-depth examination of the mechanism whereby parental social support shapes academic burnout.

The findings should be interpreted with caution because this study relied on a cross-sectional design and exclusively self-reported measures. Although Harman’s single-factor test suggested that common method bias was not a serious concern, the use of single-source self-report data may still introduce shared method variance. More importantly, the cross-sectional nature of the data does not allow for causal inferences regarding the associations among parental social support, academic self-efficacy, and academic burnout. Furthermore, while this study discussed gender differences, it did not directly assess gender-role beliefs, family expectations, or cultural value orientations. As a result, the relevant interpretations remain largely confined to the level of theoretical inference. Future research may adopt longitudinal designs, multi-source data collection, or mixed-method approaches, and further integrate variables related to gender roles and family structure, to more directly test the underlying mechanisms across diverse groups.

## 9 Conclusion

This study examined the negative association between parental social support and academic burnout among vocational undergraduate students, with academic self-efficacy serving as a mediator in this association. Furthermore, we examined whether these associations differed between male and female students. The results suggest that parental social support may be associated with lower academic burnout not only directly, but also indirectly through academic self-efficacy. In addition, the association between parental social support and academic self-efficacy was stronger among male students than among female students. Therefore, Chinese vocational undergraduate colleges should develop targeted support measures to enhance the effectiveness of educational policies aimed at reducing academic burnout. Specifically, these institutions should provide academic guidance and practical learning opportunities, such as internships and part-time job opportunities tailored to female students’ needs, to help them strengthen academic confidence, reduce financial pressure, and develop professional skills. Educational policies should also incorporate gender-sensitive strategies, such as counseling services that address the challenges faced by female students in managing academic difficulties. By focusing on these targeted support measures, educational institutions can contribute to creating a more inclusive and supportive environment for all students.

## Data Availability

The raw data supporting the conclusions of this article will be made available by the authors, without undue reservation.

## References

[ref1] Artino A. R. (2012). Academic self-efficacy: from educational theory to instructional practice. Perspect. Med. Educ. 1, 76–85. doi: 10.1007/s40037-012-0012-5, 23316462 PMC3540350

[ref2] Bandura A. (2001). Social cognitive theory: an agentic perspective. Annu. Rev. Psychol. 52, 1–26. doi: 10.1146/annurev.psych.52.1.1, 11148297

[ref3] Byrne B. M. (2010). Structural Equation Modeling with AMOS: Basic Concepts, Applications, and Programming. 2nd Edn New York, NY: Routledge.

[ref4] Charkhabi M. Abarghuei M. A. Hayati D. (2013). The association of academic burnout with self-efficacy and quality of learning experience among Iranian students. Springerplus 2:677. doi: 10.1186/2193-1801-2-677, 24386623 PMC3872283

[ref5] Chyu E. P. Y. Chen J. K. (2024). Mediating effects of different sources of perceived social support on the association between academic stress and mental distress in Hong Kong. Child Youth Serv. Rev. 163:107808. doi: 10.1016/j.childyouth.2024.107808

[ref6] Endendijk J. J. Groeneveld M. G. Mesman J. (2018). The gendered family process model: an integrative framework of gender in the family. Arch. Sex. Behav. 47, 877–904. doi: 10.1007/s10508-018-1185-8, 29549542 PMC5891573

[ref7] Garcia P. R. J. M. Restubog S. L. D. Toledano L. S. Tolentino L. R. Rafferty A. E. (2012). Differential moderating effects of student- and parent-rated support in the relationship between learning goal orientation and career decision-making self-efficacy. J. Career Assess. 20, 22–33. doi: 10.1177/1069072711417162

[ref8] Green Z. A. Çiçek İ. Yıldırım M. (2024). The relationship between social support and uncertainty of COVID-19: the mediating roles of resilience and academic self-efficacy. Psihologija 57, 407–427. doi: 10.2298/PSI220903002G

[ref9] Hair J. F. Black W. C. Babin B. J. Anderson R. E. (2010). Multivariate Data Analysis. 7th Edn Upper Saddle River, NJ: Prentice Hall, Pearson.

[ref10] Halpern H. P. Perry-Jenkins M. (2016). Parents' gender ideology and gendered behavior as predictors of children's gender-role attitudes: a longitudinal exploration. Sex Roles 74, 527–542. doi: 10.1007/s11199-015-0539-0, 27445431 PMC4945126

[ref11] Harman H. H. (1976). Modern Factor Analysis. Chicago, IL: University of Chicago Press.

[ref12] Hashmi A. Amirah N. Yusof Y. Zaliha T. (2021). Mediation of inventory control practices in proficiency and organizational performance: state-funded hospital perspective. Uncertain Supply Chain Manag 9, 89–98. doi: 10.5267/j.uscm.2020.11.006

[ref13] Huang C. (2013). Gender differences in academic self-efficacy: a meta-analysis. Eur. J. Psychol. Educ. 28, 1–35. doi: 10.1007/s10212-011-0097-y

[ref14] Huang Z. Ismail I. A. Ghazali A. H. A. D'Silva J. L. Abdullah H. Zhang Z. (2025). Uncovering a suppressor effect in the relationship between psychological capital and employment expectations: a chain mediation model among vocational undergraduates. Front. Psychol. 16:1461983. doi: 10.3389/fpsyg.2025.1461983, 40599536 PMC12209289

[ref15] Jacobs S. R. Dodd D. (2003). Student burnout as a function of personality, social support, and workload. J. Coll. Stud. Dev. 44, 291–303. doi: 10.1353/csd.2003.0028

[ref16] Joo S. H. Durband D. B. Grable J. (2008). The academic impact of financial stress on college students. J. Coll. Stud. Retent.: Res. Theory Pract. 10, 287–305. doi: 10.2190/CS.10.3.c

[ref17] Karademas E. C. (2006). Self-efficacy, social support and well-being: the mediating role of optimism. Pers. Individ. Dif. 40, 1281–1290. doi: 10.1016/j.paid.2005.10.019

[ref18] Kim B. Jee S. Lee J. An S. Lee S. M. (2018). Relationships between social support and student burnout: a meta-analytic approach. Stress. Health 34, 127–134. doi: 10.1002/smi.2771 28639354

[ref19] Labrague L. J. (2024). Examining the influence of social support and resilience on academic self-efficacy and learning outcomes in pre-licensure student nurses. J. Prof. Nurs. 55, 119–124. doi: 10.1016/j.profnurs.2024.09.012, 39667877

[ref20] Laksmiwati E. D. Tondok M. S. (2023). Perceived social support, academic self-efficacy, and anxiety among final year undergraduate students: a mediation analysis. Bull. Couns. Psychother. 5, 173–182. doi: 10.51214/00202305514000

[ref21] Lease S. H. Dahlbeck D. T. (2009). Parental influences, career decision-making attributions, and self-efficacy: differences for men and women? J. Career Dev. 36, 95–113. doi: 10.1177/0894845309340794

[ref22] Li S. Cao Z. Han T. (2023). Research and practice of training of innovative talents in vocational undergraduate education. Front Educ Res 6, 155–159. doi: 10.25236/FER.2023.062726

[ref23] Liu X. Sun X. Hao Q. (2022). Influence of discrimination perception on career exploration of higher vocational students: chain mediating effect test. Front. Psychol. 13:968032. doi: 10.3389/fpsyg.2022.968032, 35967637 PMC9363697

[ref24] Liu Z. Xie Y. Sun Z. Liu D. Yin H. Shi L. (2023). Factors associated with academic burnout and its prevalence among university students: a cross-sectional study. BMC Med. Educ. 23:317. doi: 10.1186/s12909-023-04316-y, 37149602 PMC10163855

[ref25] Louie V. (2001). Parents' aspirations and investment: the role of social class in the educational experiences of 1.5- and second-generation Chinese Americans. Harv. Educ. Rev. 71, 438–475. doi: 10.17763/haer.71.3.lv51475vjk600h38

[ref26] Lu Y. Wang T. (2023). Quality evaluation model of vocational education in China: a qualitative study based on grounded theory. Educ. Sci. 13:819. doi: 10.3390/educsci13080819

[ref27] Ma Y. (2024). The impact of academic self-efficacy and academic motivation on Chinese EFL students' academic burnout. Learn. Motiv. 85:101959. doi: 10.1016/j.lmot.2024.101959

[ref28] Mazzuca C. Majid A. Lugli L. Nicoletti R. Borghi A. M. (2020). Gender is a multifaceted concept: evidence that specific life experiences differentially shape the concept of gender. Lang. Cogn. 12, 649–678. doi: 10.1017/langcog.2020.15

[ref29] Murphy R. Tao R. Lu X. (2011). Son preference in rural China: patrilineal families and socioeconomic change. Popul. Dev. Rev. 37, 665–690. doi: 10.1111/j.1728-4457.2011.00452.x, 22319769

[ref30] Narayanan S. S. Onn A. C. W. (2016). The influence of perceived social support and self-efficacy on resilience among first year Malaysian students. Kajian Malaysia 34, 1–23. doi: 10.21315/km2016.34.2.1

[ref31] Nielsen T. Dammeyer J. Vang M. L. Makransky G. (2018). Gender fairness in self-efficacy? A Rasch-based validity study of the general academic self-efficacy scale (GASE). Scand. J. Educ. Res. 62, 664–681. doi: 10.1080/00313831.2017.1306796

[ref32] Paulssen M. Roulet R. Wilke S. (2014). Risk as moderator of the trust-loyalty relationship. Eur. J. Mark. 48, 964–981. doi: 10.1108/EJM-11-2011-0657

[ref33] Procidano M. E. Heller K. (1983). Measures of perceived social support from friends and from family: three validation studies. Am. J. Community Psychol. 11, 1–24. doi: 10.1007/BF00898416, 6837532

[ref34] Qing S. (2020). Gender role attitudes and male-female income differences in China. J. Chin. Sociol. 7:12. doi: 10.1186/s40711-020-00123-w

[ref35] Rahman M. S. Fattah F. A. M. A. Zaman M. Hassan H. (2018). Customer's patronage decision toward health insurance products: mediation and multi-group moderation analysis. Asia Pac. J. Mark. Logist. 30, 62–83. doi: 10.1108/APJML-12-2016-0248

[ref36] Rahmati Z. (2015). The study of academic burnout in students with high and low level of self-efficacy. Procedia. Soc. Behav. Sci. 171, 49–55. doi: 10.1016/j.sbspro.2015.01.087

[ref37] Ren Z. Hu L. Yu J. J. Yu Q. Chen S. Ma Y. . (2020). The influence of social support on physical activity in Chinese adolescents: the mediating role of exercise self-efficacy. Children 7:23. doi: 10.3390/children7030023, 32245103 PMC7140834

[ref38] Ryan R. M. Deci E. L. (2000). Self-determination theory and the facilitation of intrinsic motivation, social development, and well-being. Am. Psychol. 55, 68–78. doi: 10.1037/0003-066X.55.1.68, 11392867

[ref39] Saefudin W. Sriwiyanti S. Yusoff S. H. M. (2021). Role of social support toward student academic self-efficacy in online learning during pandemic. Jurnal Tatsqif 19, 133–154. doi: 10.20414/jtq.v19i2.4221

[ref40] Schaufeli W. B. Martinez I. M. Pinto A. M. Salanova M. Bakker A. B. (2002). Burnout and engagement in university students: a cross-national study. J. Cross-Cult. Psychol. 33, 464–481. doi: 10.1177/0022022102033005003

[ref41] Singh L. B. Kumar A. Srivastava S. (2021). Academic burnout and student engagement: a moderated mediation model of internal locus of control and loneliness. J. Int. Educ. Bus. 14, 219–239. doi: 10.1108/JIEB-03-2020-0020

[ref42] Streiner D. L. (2003). Starting at the beginning: an introduction to coefficient alpha and internal consistency. J. Pers. Assess. 80, 99–103. doi: 10.1207/S15327752JPA8001_18, 12584072

[ref43] Tang S. Osman S. Z. M. (2023). Higher vocational college students' learning burnout during the COVID-19 pandemic: a case study in China. Int. J. Eval. Res. Educ. 12, 684–691. doi: 10.11591/ijere.v12i2.24057

[ref44] Torkzadeh G. Koufteros X. Pflughoeft K. (2003). Confirmatory analysis of computer self-efficacy. Struct. Equ. Model. 10, 263–275. doi: 10.1207/S15328007SEM1002_6

[ref45] Wang S. (2023). Exploration of undergraduate vocational education in China: process, experience and strategy. J. Educ. Train. Stud. 11, 83–98. doi: 10.11114/jets.v11i4.6426

[ref46] Wang Y. Chung M. C. Wang N. Yu X. Kenardy J. (2021). Social support and posttraumatic stress disorder: a meta-analysis of longitudinal studies. Clin. Psychol. Rev. 85:101998. doi: 10.1016/j.cpr.2021.101998, 33714168

[ref47] Wang W. Liu X. Dong Y. Bai Y. Wang S. Zhang L. (2020). Son preference, eldest son preference, and educational attainment: evidence from Chinese families. J. Fam. Issues 41, 636–666. doi: 10.1177/0192513X19874091

[ref48] Xia Q. Pang C. Zeng W. Li Q. Wu S. Xiao X. (2025). Burnout among VET pathway university students: the role of academic stress and school-life satisfaction. BMC Public Health 25:1467. doi: 10.1186/s12889-025-22769-2, 40259307 PMC12010583

[ref49] Xiong J. (2011). Understanding higher vocational education in China: vocationalism vs Confucianism. Front. Educ. China 6, 495–520. doi: 10.1007/s11516-011-0143-1

[ref50] Yang D. Tu C. C. Guo Z. Dai X. Tu C. F. (2023). Moderating mechanism in the relationship between social isolation and mental health among college students during high-risk period of COVID-19 transmission in Hubei, China. Int. J. Ment. Health Promot. 25, 193–206. doi: 10.32604/ijmhp.2022.022130

[ref51] Ye Y. Huang X. Liu Y. (2021). Social support and academic burnout among university students: a moderated mediation model. Psychol. Res. Behav. Manag. 14, 335–344. doi: 10.2147/PRBM.S300797, 33776493 PMC7987309

[ref52] Ye Y. Wang C. Zhu Q. He M. Havawala M. Bai X. . (2022). Parenting and teacher-student relationship as protective factors for Chinese adolescent adjustment during COVID-19. Sch. Psychol. Rev. 51, 187–205. doi: 10.1080/2372966X.2021.1897478

[ref53] Yeh Y. C. Ko H. C. Wu J. Y. W. Cheng C. P. (2008). Gender differences in relationships of actual and virtual social support to internet addiction mediated through depressive symptoms among college students in Taiwan. Cyberpsychol. Behav. 11, 485–487. doi: 10.1089/cpb.2007.0134, 18721099

[ref54] Zhang Y. Guo H. Ren M. Ma H. Chen Y. Chen C. (2024). The multiple mediating effects of self-efficacy and resilience on the relationship between social support and procrastination among vocational college students: a cross-sectional study. BMC Public Health 24:1958. doi: 10.1186/s12889-024-19487-6, 39039457 PMC11264396

[ref55] Zhang Y. Jin S. (2016). The impact of social support on postpartum depression: the mediator role of self-efficacy. J. Health Psychol. 21, 720–726. doi: 10.1177/1359105314536454, 24925546

[ref56] Zhang J. Meng J. Wen X. (2025). The relationship between stress and academic burnout in college students: evidence from longitudinal data on indirect effects. Front. Psychol. 16:1517920. doi: 10.3389/fpsyg.2025.1517920, 40491945 PMC12146318

[ref57] Zhu Y. Lu H. Wang X. Ma W. Xu M. (2025). The relationship between perceived peer support and academic adjustment among higher vocational college students: the chain mediating effects of academic hope and professional identity. Front. Psychol. 16:1534883. doi: 10.3389/fpsyg.2025.1534883, 40062197 PMC11885234

[ref58] Zimmerman B. J. (2000). Self-efficacy: an essential motive to learn. Contemp. Educ. Psychol. 25, 82–91. doi: 10.1006/ceps.1999.1016, 10620383

[ref59] Zosuls K. M. Miller C. F. Ruble D. N. Martin C. L. Fabes R. A. (2011). Gender development research in sex roles: historical trends and future directions. Sex Roles 64, 826–842. doi: 10.1007/s11199-010-9902-3, 21747580 PMC3131694

